# Efficient Selection of Recombinant Fluorescent Vaccinia Virus Strains and Rapid Virus Titer Determination by Using a Multi-Well Plate Imaging System

**DOI:** 10.3390/biomedicines9081032

**Published:** 2021-08-18

**Authors:** Mingyu Ye, Markus Keicher, Ivaylo Gentschev, Aladar A. Szalay

**Affiliations:** 1 Department of Biochemistry and Cancer Therapy Research Center (CTRC), Biocenter, University of Wuerzburg, Theodor-Boveri-Weg 1, 97074 Wuerzburg, Germany; markus.keicher@stud-mail.uni-wuerzburg.de (M.K.); ivaylo.gentschev@mail.uni-wuerzburg.de (I.G.); 2 Department of Radiation Oncology, Rebecca & John Moores Comprehensive Cancer Center, University of California, San Diego, CA 92093, USA; 3 Department of Pathology, Center of Immune Technologies, Stanford University School of Medicine, Stanford, CA 94305, USA

**Keywords:** fluorescent recombinant vaccinia virus, plaque isolation, IncuCyte^®^S3, plaque assay

## Abstract

Engineered vaccinia virus (VACV) strains are used extensively as vectors for the development of novel cancer vaccines and cancer therapeutics. In this study, we describe for the first time a high-throughput approach for both fluorescent rVACV generation and rapid viral titer measurement with the multi-well plate imaging system, IncuCyte^®^S3. The isolation of a single, well-defined plaque is critical for the generation of novel recombinant vaccinia virus (rVACV) strains. Unfortunately, current methods of rVACV engineering via plaque isolation are time-consuming and laborious. Here, we present a modified fluorescent viral plaque screening and selection strategy that allows one to generally obtain novel fluorescent rVACV strains in six days, with a minimum of just four days. The standard plaque assay requires chemicals for fixing and staining cells. Manual plaque counting based on visual inspection of the cell culture plates is time-consuming. Here, we developed a fluorescence-based plaque assay for quantifying the vaccinia virus that does not require a cell staining step. This approach is less toxic to researchers and is reproducible; it is thus an improvement over the traditional assay. Lastly, plaque counting by virtue of a fluorescence-based image is very convenient, as it can be performed directly on the computer.

## 1. Introduction

Vaccinia virus (VACV) is a double-stranded DNA virus belonging to the Poxviridae family. In the past, it was utilized as a vaccine against smallpox, and it has been widely researched in the context of molecular biology and pathogenesis [1]. In recent decades, VACV has been used extensively as a vector for the treatment of patients with advanced-stage solid cancers [2]. VACV is very promising with regard to its use as an oncolytic agent. This is mainly because of its safety, large foreign DNA size capacity (up to 25 kb), and natural tumor tropism [3,4,5,6]. Recombinant virus engineering is one of the most fundamental experimental techniques in VACV research [7,8,9]. The traditional way to generate recombinant vaccinia virus (rVACV) strains is based on homologous recombination (HR) events that occur between the VACV genome and homologous sequences in the shuttle vector [10]. However, recombination occurs very infrequently; typically, only 1–5% of the viral plaques will contain the given gene fragment. This makes it difficult to screen for positive recombinant viral plaques [11]. To make the creation of rVACV easier and faster, there are two main options: increase the homologous recombination (HR) rate or enhance the efficiency of positive plaque purification. Ming Yuan et al., using the CRISPR-Cas9 gene-editing system, developed an efficient method of VACV gene modification, which greatly increases the recombination rate via induction of viral genome double-strand breaks (DSBs), making the positive plaques easily identifiable, i.e., marked, in the first round of purification [12,13]. However, both the efficiency of DSB formation and the break site in the viral genome are synthetic single-guide RNA (sgRNA) dependent. Therefore, when constructing a new strain of rVACV using the CRISPR-Cas9 system, the additional steps of gRNA synthesis and screening are required. Furthermore, in some cases, CRISPR-Cas9 gene editing can cause large deletions and complex rearrangements in the genome [14]. To increase the efficiency of rVACV positive plaque purification, Brittany Jasperse et al. recently developed a novel platform called “Efficient Purification by Parental Inducer Constraint” (EPPIC). The EPPIC platform allows for the generation and purification of rVACV with or without markers in six days [15]. Nevertheless, this platform not only requires a replication-inducible parental virus containing an inducible element but also a specific drug to induce viral replication [15]. Zong Sheng Guo et al. used fluorescence-activated cell sorting (FACS) to collect and plate single YFP-positive cells which may be infected with rVACV into a CV-1 cell-seeded 96-well plate in order to purify fluorescence-labeled rVACV. This drug-free selection system significantly accelerated the generation of fluorescent rVACV while minimizing the viral mutation rate in the absence of the drug-mediated selective pressure [16]. However, employing a FACS machine and sorting procedure for the purification of virally infected cells is somewhat costly, especially for two rounds of purification, and the manipulation of a FACS machine requires a highly trained specialist. In addition, one or two rounds of plaque purification are still essential for some clones even after two rounds of FACS [16]. Therefore, the process of rVACV construction should continue to be optimized. In this study, we present a modified, novel method for the generation and purification of engineered vaccinia virus strains by using green or red fluorescent protein-encoding genes in combination with the multi-well imaging system IncuCyte S3 (Essen BioScience, Royston, UK, Cat. no.: 4647).

Virus titer determination is one of the most critical procedures in virology research and viral quantification of clinical samples [17,18]. The traditional methods for measuring viral titers are the plaque assay and the fifty-percent tissue culture infective dose (TCID50) assay [19,20]. Both of these techniques are based on the ability to quantify visible cytopathic effects (CPEs) in vitro (expressed either as PFU or TCID50), which requires a cell monolayer to be infected and lysed by the virus of interest with serial dilutions [21,22,23]. For VACV titer determination, the plaque assay is the most common method. The viral plaque is an area in a cell monolayer that displays a cytopathic effect, i.e., after the cells are infected [24]. Each plaque represents a single infectious unit. By staining the cellular layer with chemical dyes, the plaques appear as distinguishable spots, as their centers are devoid of cells because of viral infection. In the typical viral plaque assay, a confluent monolayer of the host cells is first infected with the virus at different dilutions [25,26]. Then, the infected cell monolayer is covered with a solid (e.g., agarose) or semi-solid (e.g., methylcellulose) overlay medium to prevent the progeny virus from spreading through the medium [27]. After a specific incubation period (which depends on the type of virus), the infected cells are lysed via viral replication, and individual plaques are formed [28]. To visualize the viral plaques for counting purposes, the cell monolayer is fixed and stained with chemical dyes such as neutral red, 3-(4,5-dimethylthiazol-2-yl)-2,5-diphenyl tetrazolium bromide (MTT), or crystal violet solution [29]. Plaques are generally counted manually and used to calculate the number of plaque-forming units per sample unit volume (pfu/mL). In this work, we outline a novel approach for the plaque assay that involves the multi-well plate imaging system IncuCyte^®^S3 and does not require the traditional cell fixation and staining steps. The IncuCyte^®^S3 is a machine that can provide instant and real-time cell images (including whole well cell images) and thereby help to derive more profound and physiologically relevant information about the cells, such as confluence and fluorescence intensity [30].

In summary, using a multi-well plate imaging system (IncuCyte^®^S3), we designed a new experimental system that can significantly accelerate the process of fluorescent rVACV generation and VACV viral titer determination.

## 2. Materials and Methods

### 2.1. Cell Lines

**Figure A1:**
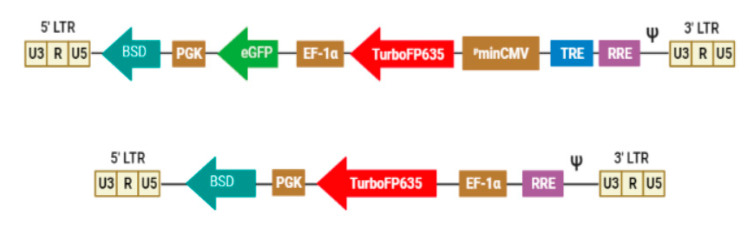
Stable cell lines constructed by lentiviral vector: **A1:** CV-1-pTet-TurboFP635-EF-1a-Egfp cell line. **A2:** CV-1-EF-1a-TurboFP635 cell line.

The African green monkey kidney fibroblast cell line (CV-1) was obtained from the American Type Culture Collection (ATCC; CCL-70). The stable cell lines CV-1-pTet-TurboFP635-EF-1a-Egfp and CV-1-EF-1a-TurboFP635 (Figure A1, Appendix A) were generated and propagated in Dulbecco’s modified Eagle’s medium (DMEM; 11965092, Thermo Fisher Scientific) supplemented with 10% fetal bovine serum (FBS, F4135, Sigma) and 1% penicillin–streptomycin (P4333, Sigma).

### 2.2. Vaccinia Virus Strains 

Vaccinia virus strain LIVP 1.1.1 was derived from LIVP (Lister strain, Institute of Viral Preparations, Moscow, Russia) [31]. Vaccinia virus strain GLV-1h109 [32] was constructed from the oncolytic vaccinia virus GLV-1h68 [33] by inserting the *glaf-1* gene encoding the GLAF-1 antibody under the control of the vaccinia virus promoter (p7.5) into the J2R locus. L1c-Ig-Turbo is derived from LIVP1.1.1-strain in which the TurboPF635 gene was inserted in the space between the intergenic locus 157 and intergenic locus 158. L1c-Ig-Turbo-TK-Egfp was created by inserting the eGFP gene under the control of the p7.5 promoter into the J2R locus of L1c-Ig-Turbo (this study). 

### 2.3. Plasmid

To construct the pSC65-eGFP shuttle vector, the LacZ-encoding fragment was deleted in the pSC65 plasmid by double digestion with Xho I and EcoR I restriction enzymes and replaced with the eGFP gene. 

### 2.4. Recombinant Vaccinia Virus Generation by Homologous Recombination 

Day 1: Seed 3–4 × 10^5^ CV-1 cells with 2 mL complete growth medium per well in a 6-well plate to reach 80% confluence the next day. Day 2: Aspirate the medium from one well of the plate, then infect the cells with 4 × 10^4^ pfu L1c-Ig-Turbo virus and put the plate into the CO_2_ incubator for 2 h (agitate the plate every 30 min). Transfect 1 µg pmaxGFP plasmid with 6 µL TurboFectin 8.0 (TF81001, ORIGENE) into virus-uninfected wells and 3–4 µg pSC65-eGFP donor plasmid with 24 µL TurboFectin 8.0 into the virus-infected wells, put the plate into the CO_2_ incubator for another two days, then use the IncuCyte^®^S3 whole well imaging program to check the fluorescence signal to evaluate the transfection efficiency. Day 4: Aspirate the medium and use the scraper to harvest the infected cells in 1 mL FBS-free DMEM (frozen in −80 °C for backup use) as a stock for further plaque screening; meanwhile, seed 4–5 × 10^4^ CV-1 cells with 100 µL complete growth medium per well into six 96-well plates to obtain 100% confluence the next day. Day 5: Process the viral stock from the former step with a sonicator (sonicate 20 to 30 sec at full power), vortex the stock and add 0.25 µL liquid into the 61 mL FBS-free DMEM, then use the multi-channel pipette to add 100 µL virus-containing DMEM per well into the six 96-well plates. Put all the 96-well plates into the IncuCyte^®^S3 to set up a scan schedule that will initiate the plate scanning after 1.5–2 days. After all the plates are scanned, suspend the scheduled program. Check the images and mark the plaques under the standard optical microscope, then pick out the positive clones with pipette tips in 200 µL DMEM/PBS for a second round of purification (we suggest using 6- or 24-well plates for the next round of purification) and purify the recombinant virus until negative plaques are no longer observed.

### 2.5. Fluorescence-Based Viral Plaque Assay

Day 1: Seed 6 × 10^5^ of each cell line (wild-type CV-1, CV-1-pTet-TurboFP635-EF-1a-Egfp, and CV-1-EF-1a-TurboFP635) with 2 mL 10% FBS DMEM per well in a 6-well plate to reach 100% confluence the next day. Day 2: Thaw an aliquot of vaccinia virus and sonicate at full power for 1 min in ice water (repeated three times), then prepare dilutions for each viral strain (Lister 1.1.1, L1c-Ig-Turbo, and GLV-1h109) as Table S1 shows. Remove the medium from the cell culture plates, infect the cells with 250 µL viral solution from dilution 10–5 to 10–7 in duplicate, incubate the plate at 37 °C in the CO_2_ incubator for 2 h, and gently shake the plate every 20 min. Aspirate the infection media, add 2 mL of 5% FBS CMC culture media and incubate another for two days for further analysis. Day 4: For the image visualization, the plates are directly scanned on green or red fluorescence channels using a whole well image program via the Incucyte^®^S3. The cell monolayer is photographed at high resolution. Then, use the Incucyte^®^S3 self-contained software to process the image and remove the background to obtain a distinct picture. After image visualization, the pictures are imported into the FreeCAD software for plaque counting. In the case of low virus concentration wells, the number of viral plaques can be determined directly from the image created by the Incucyte^®^S3.

### 2.6. Viral Plaque Assay for Statistical Analysis

Day 1: Seed 6 × 10^5^ of each cell line (wild-type CV-1, CV-1-pTet-TurboFP635-EF-1a-Egfp, and CV-1-EF-1a-TurboFP635) with 2 mL 10% FBS DMEM per well in a 6-well plate to reach 100% confluence the next day. Day 2: Thaw an aliquot of vaccinia virus Lister 1.1.1 and sonicate at full power for 1 min in ice water (repeated three times), and dilute the virus to 10–6. Then, remove the medium from the cell culture wells, infect the cells with 250 µL viral solution per well, incubate the plate at 37 °C, 5% CO_2_ for 2 h, and gently shake the plate every 20 min. Aspirate the infection media, add 2 mL of 5% FBS CMC culture media and incubate at 37 °C, 5% CO_2_ for another two days. Day 4: Remove the CMC medium, stain the plates with 1 mL per well crystal violet for 3–5 h/overnight at room temperature, remove the stained medium into an extra glass bottle (for later sterilization) and wash the cells with tap water, then count the number of viral plaques.

### 2.7. Statistical Analysis

All the data analyses were performed, and graphics were made using GraphPad Prism 8.0 software. Statistical significance was determined by unpaired Student’s *t*-tests. Results are presented as mean ± SD (*, *p* < 0.05; **, *p* < 0.01; ***, *p* < 0.001; ****, *p* < 0.0001; ns, not significant). To ensure reproducibility, the recombinant vaccinia virus purification experiment was repeated twice, and the virus titer determination experiment was repeated three times. 

### 2.8. Software

FreeCAD software (https://www.freecadweb.org/) was used for viral plaque counting; Graphpad Prism 8.0 (https://www.graphpad.com/scientific-software/prism/) was used for statistical analyses; Incucyte^®^S3 Software (v2018B, https://www.essenbioscience.com/en/products/software/incucyte-s3-software-v2018b/) was also used.

## 3. Results

### 3.1. Generation, Selection, and Purification of the New L1c-Ig-Turbo-TK-eGFP Strain Expressing eGFP and Turbofp635 Proteins

**Figure 1:**
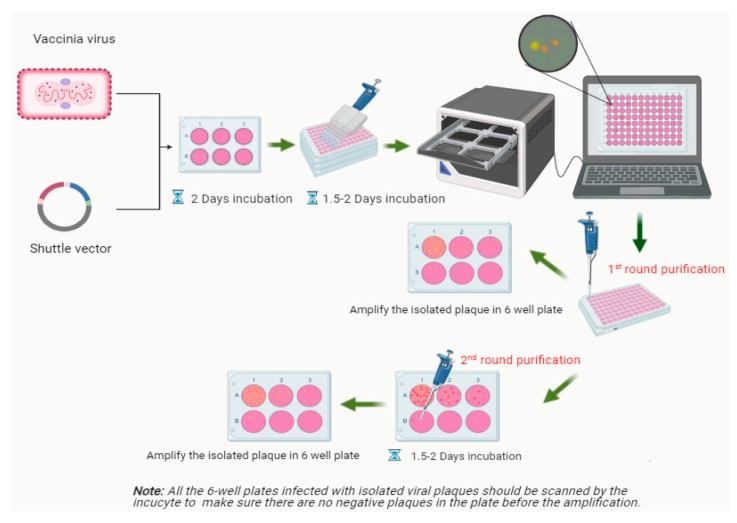
Workflow of construction and isolation of rVACV expressing fluorescent proteins.

**Figure 2:**
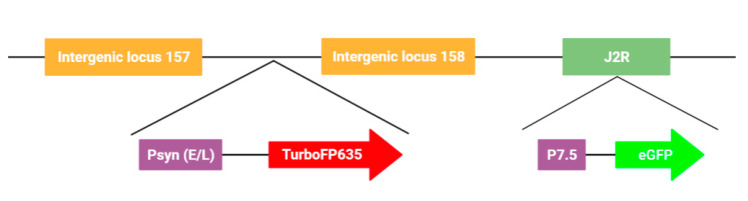
Schematic representation of recombinant vaccinia virus L1c-Ig-Turbo-TK-eGFP.

**Figure 3:**
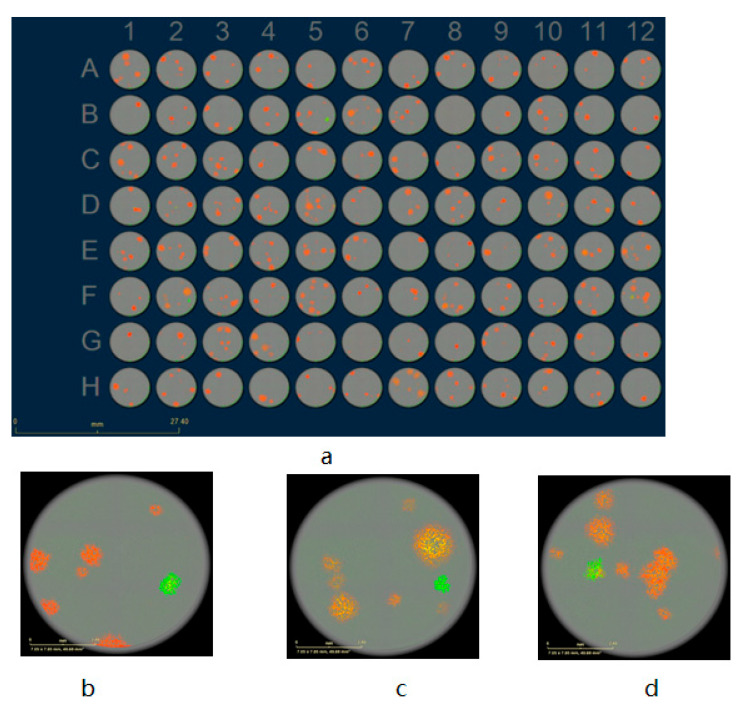
The first round of rVACV L1c-Ig-Turbo-TK-eGFP plaque selection (**a**–**d**). (**a**) Fluorescent image of the recombinant virus-infected CV-1 cells in 96-well plates after two days of incubation scanned by the IncuCyte^®^S3, scale bar = 27.4 mm. (**b**) Image enlargement (IE) from (**a**) B5 well. (**c**) IE from (**a**) F2 well. (**d**) IE from (**a**) F12 well. (**b**–**d**) The scale bars represent 2.4 mm.

**Figure 4:**
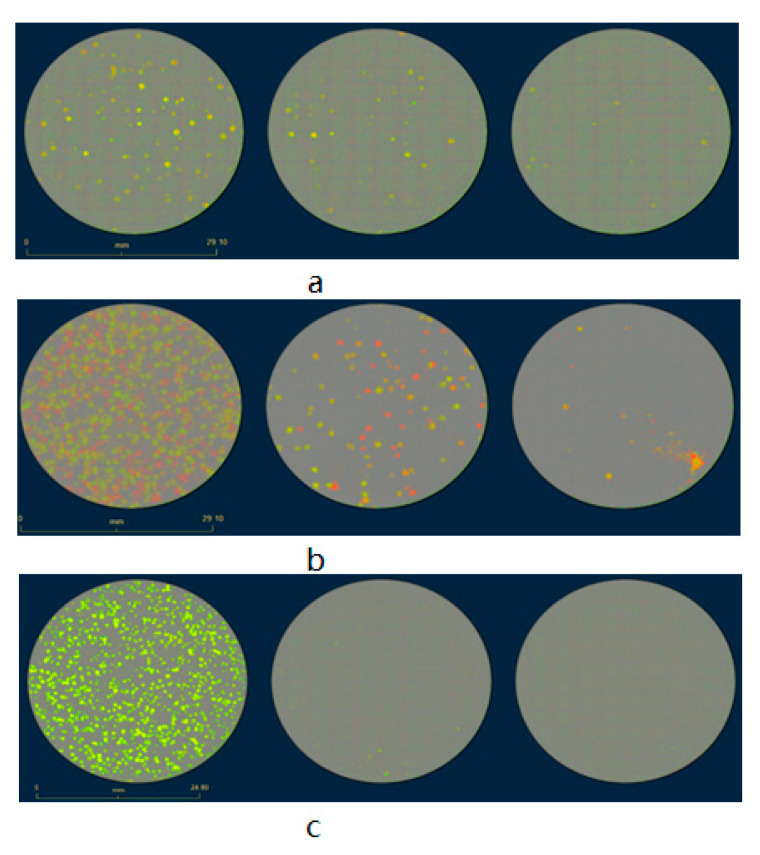
Isolation procedure of rVACV L1c-Ig-Turbo-TK-eGFP plaques with the fluorescence imaging system, IncuCyte^®^S3. Wild-type CV-1 cells were seeded in the 6-well plates, then infected with the recombinant virus mixture from left well to right well with 10-fold serial dilution; after 36–48 h of incubation, the wells were scanned by the IncuCyte^®^S3. (**a**) Plaque purification only needs to be processed with one round after picking out from a 96-well plate, scale bar = 29.10 mm. (**b**,**c**) Plaque purification needs to be processed with two rounds after picking out from a 96-well plate. (**b**) The first-round plaque purification, which still has many RFP-positive only plaques in the wells, scale bar = 29.10 mm. (**c**) The second round of purification. First, isolation of an eGFP-positive plaque from (**b**), then infection of CV-1 cells in a new 6-well plate with 10-fold serial dilution, scale bar = 24.80 mm.

The rVACV generation and purification procedures are time-consuming and cumbersome, mainly due to the requirement of many rounds of plaque purification [10]. Here, we present a new screening routine based on a multi-well plate imaging system, IncuCyte^®^S3, which could help to generate and purify fluorescent rVACV in a short period of time. The steps of this procedure are detailed in Figure 1. To estimate the viral HR rate and visualize the virus distribution in the cell culture plates, we first constructed a new rVACV strain, L1c-Ig-Turbo (Figure S1). To present the whole procedure of the modified method, the rVACV strain L1c-Ig-Turbo-TK-eGFP (Figure 2) was constructed by using shuttle vector pSC65-eGFP to insert the eGFP gene into the thymidine kinase (TK) locus of L1c-Ig-Turbo. The pSC65-eGFP shuttle plasmid was transfected into CV-1 cells, which were seeded in a 6-well plate; the plate was incubated at 37 °C, 5% CO2 for two hours. After two hours of incubation, the transfected cells were infected with the L1c-Ig-Turbo virus for another two days of incubation. Then, the plates were scanned by the IncuCyte^®^S3 (Figure S2), and the well infected with the virus was harvested as a stock for further selection. For the first round of rVACV plaque isolation and purification, portions of the viral stock were diluted and infected with the CV-1 cells that were seeded in six 96-well plates. After 1.5–2 days of incubation, all the plates were scanned and imaged by the IncuCyte^®^S3 again (Figure 3). The HR rates of different plates are shown in Table 1. The positive plaques were picked out and collected by tips according to the images created from the first round of purification. For the second round of purification, the collected plaques from the first round were gradient diluted and infected the CV-1 cells in 6- or 24-well plates. After another 1.5–2 days of incubation, the plates were scanned by the IncuCyte^®^S3, and the new plaques were selected with pipette tips (the purification should be continued until there are no negative plaques left). Finally, the purified rVACV plaques were amplified as a viral stock. To analyze the efficiency of this method of rVACV selection and purification, we randomly picked out 12 positive viral plaques from the 96-well plates. Further analysis demonstrated that five of them only needed one round of plaque purification and the other seven clones needed two rounds (Figure 4).

### 3.2. Vaccinia Virus Titer Determination Based on Fluorescence-Dependent Plaque Assay

**Figure 5:**
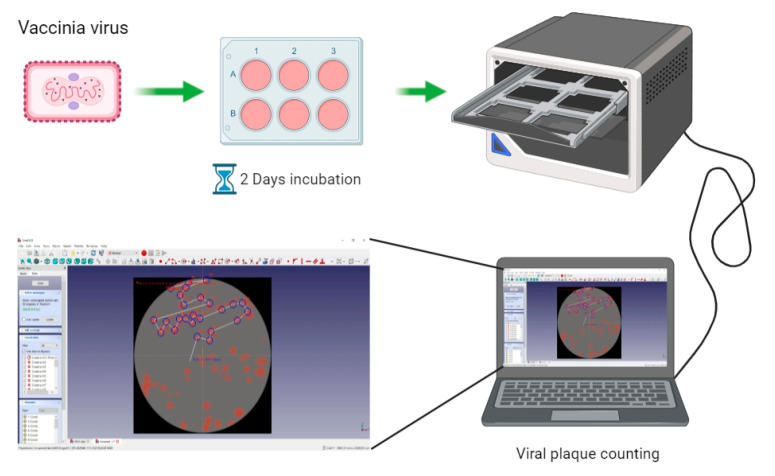
Workflow of VACV titer determination based on the fluorescence-dependent plaque assay.

**Figure 6:**
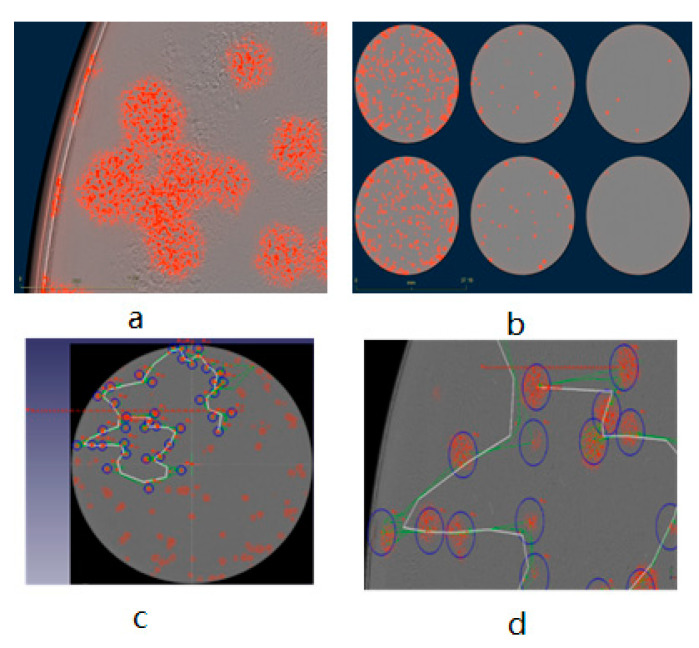
Fluorescence-dependent plaque assay. TurboFP635-labeled rVACV plaque assay based on wild-type CV-1 cells (**a**,**b**). (**a**) IE from (**b**) A1 well, scale bar = 2.3 mm. (**b**) Recombinant vaccinia virus L1c-Ig-Turbo-infected wild-type CV-1 cells, scale bar = 37.1 mm. Fluorescence-labeled viral plaques counted through FreeCAD software (**c**,**d**). (**c**) Viral plaque counting with L1c-Ig-Turbo-infected wild-type CV-1 cells through the FreeCAD software. (**d**) IE from (**c**).

The plaque assay is the gold standard for determining the infectious titer of VACV. In the traditional plaque assay, the cellular monolayer must be fixed and stained by chemical dyes after removing the immobilizing overlay medium in order to prevent indiscriminate viral spreading caused by fluid movement in the culture vessels [34]. Here, we show a fluorescence-dependent plaque assay for VACV titer determination, which is free of chemical fixation and staining steps. We further demonstrate that our modified methodology is functional, stable, and efficient. The steps of the fluorescence-dependent plaque assay are detailed in Figure 5. In this modified plaque assay, after infection of CV-1 cells with TurboFP635 fluorescent rVACV, the plaque phenotypes were analyzed by the Incucyte^®^S3, and the images with the fluorescent viral plaques were then imported into and directly counted in the FreeCAD software (Figure 6) (the data for eGFP fluorescent rVACV are shown in Figure S3). To evaluate the precision of the new counting method, we compared the data collected from the modified counting method with the data obtained from the manual crystal violet plaque counting method that involves visual inspection. The results demonstrated there are no significant differences between the plaque numbers determined by these two methods (Figure S4).

### 3.3. Development of a Plaque Assay for Non-Fluorescent rVACV on the Basis of Cell Lines Expressing Fluorescent Proteins

**Figure 7:**
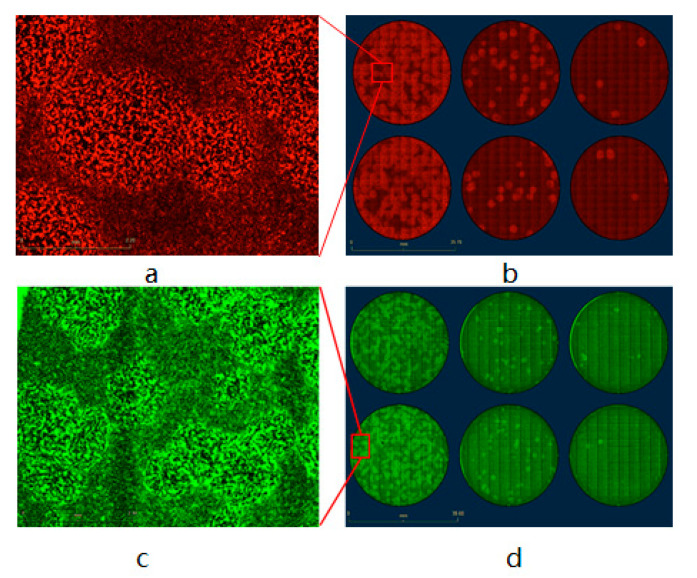
Vaccinia virus plaque assay based on fluorescent CV-1 cells (**a**–**d**). (**a**) IE from (**b**) A1 well, scale bar = 2.2 mm. (**b**) Recombinant vaccinia virus L1c-Ig-Turbo-infected wild-type CV-1 cells, scale bar = 37.7 mm. (**c**) IE from (**d**) B1 well, scale bar = 2.2 mm. (**d**) Recombinant vaccinia virus GLV-1h109-infected wild-type CV-1 cells, scale bar = 38.6 mm.

**Figure 8:**
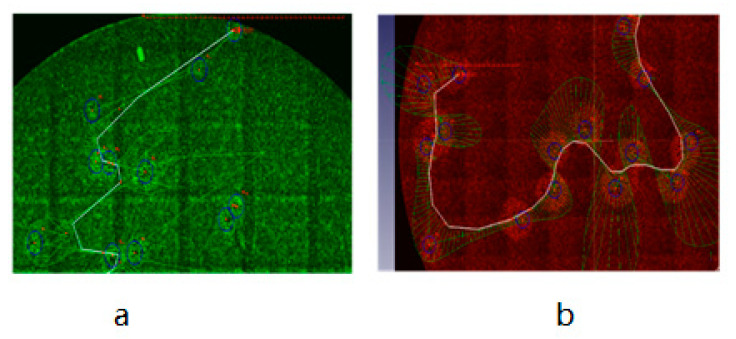
Wild-type viral plaques counted through FreeCAD software (**a**,**b**). (**a**) Viral plaque counting with LIVP1.1.1-infected CV-1-pTet-TurboFP635-EF-1a-Egfp cell line through the FreeCAD software. (**b**) Viral plaque counting with LIVP1.1.1-infected CV-1-EF-1a-TurboFP635 cell line through the FreeCAD software.

**Figure 9:**
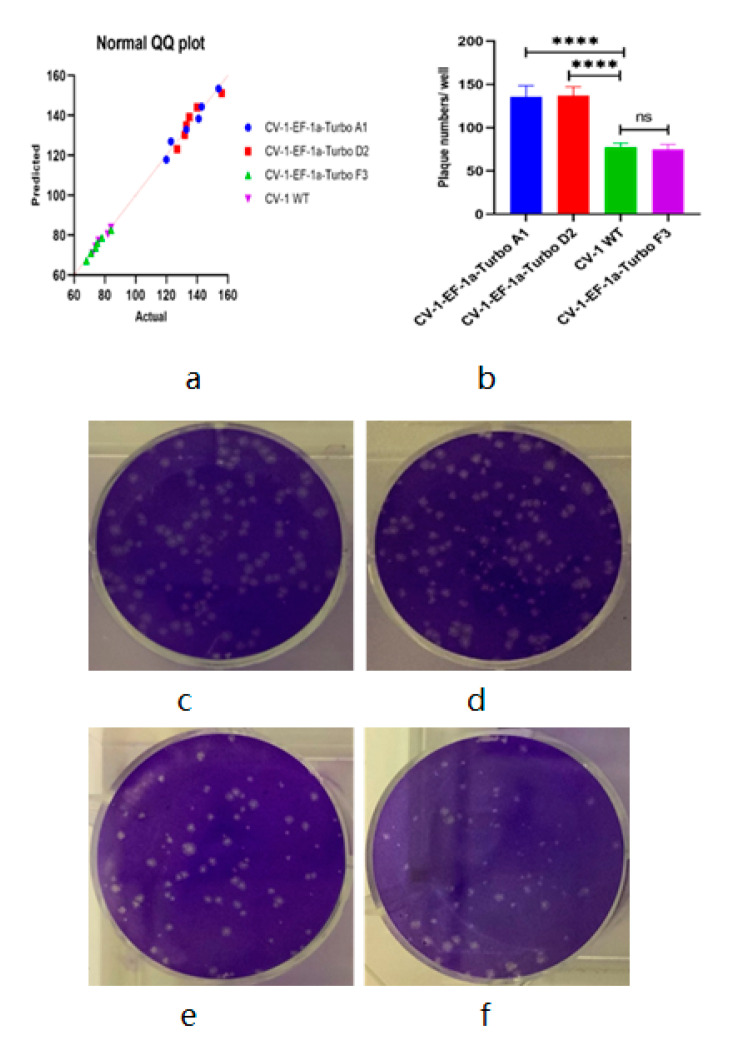
Statistical comparison of viral plaque counting using different CV-1 cell lines as the host. (**a**) Q–Q plot of plaque counts using different CV-1 cell lines. (**b**) Statistical comparison of plaque counts using different CV-1 cell lines through crystal violet staining method (*n* = 6). ns: not significant, *p* = 0.4509; **** *p* < 0.0001 (two-way ANOVA 6 SEM). Crystal violet stain of different Lister 1.1.1-infected CV-1 cell lines (**c**–**f**). (**c**) Lister 1.1.1-infected CV-1-EF-1a-TurboFP635-A1 cell line. (**d**) LIVP 1.1.1-infected CV-1-EF-1a-TurboFP635-D2 cell line. (**e**) LIVP 1.1.1-infected wild-type CV-1 cells. (**f**) LIVP 1.1.1-infected CV-1-EF-1a-TurboFP635-F3 cell line.

For the plaque assay of non-fluorescent VACV, first, we constructed two strains of eGFP- and TurboFP635-expressing CV-1 stable cell lines as described in the Materials and Methods section, then infected the fluorescent-labeled CV-1 cells with vaccinia virus strain LIVP 1.1.1 in the 6-well plate. After two hours of incubation, the infection media was aspirated and replaced with DMEM 5% FBS CMC culture media, incubated for another two days, and finally the 6-well plates were scanned in the IncuCyte^®^S3 to generate the images. The results showed that both eGFP and TurboFP635 stable cell lines could successfully form visible fluorescent viral plaques (Figure 7 and Figure 8) after being scanned by the IncuCyte^®^S3. For precise analysis, we used three different TurboFP635-expressing monoclonal CV-1 stable cell lines as the host cells for the plaque assay. After infecting these three stable cell lines with the same concentration of the Lister 1.1.1 virus, the plates were incubated for two days under the CMC overlay media. Then, the number of viral plaques was counted after crystal violet staining. In these experimental settings, we found that the plaque numbers generated by monoclonal cell line strain F3 are not significantly different from the wild-type CV-1 cell line (Figure 9). Interestingly, after non-fluorescent rVACV infection, the fluorescence intensity of the infected cells in the formed plaques is much brighter than that of uninfected neighbor cells. One possible explanation could be that the VACV infection triggered cell–cell fusion, lead to the virus-infected cells with more abundant fluorescent proteins than the uninfected cells, and therefore the plaques could be directly identified (Figure S5). Taken together with the fluorescent VACV plaque assay in the previous part, our data demonstrated that the fluorescence-dependent plaque assay method for VACV titer determination is functional and efficient.

## 4. Discussion

The low frequency of homologous recombination makes editing of VACV genomes inefficient in that it is inconvenient to screen out the positive viral plaques in the plate in the first round of selection. Eileen Nakano et al. reported that, under optimal conditions, recombinant viruses might be generated at a frequency of 1% to 5% [11]. Indeed, in our study, the homologous recombination has proven to be more inefficient, with only around a 1% recombination rate (Table 1). This fact causes extreme difficulty for rVACV purification. To improve the efficiency of fluorescent rVACV construction when using the standard HR system, we developed a new fluorescent plaque screening strategy based on a multi-well plate imaging system, IncuCyte^®^S3. Our new method significantly improved the efficiency of the whole plaque isolation process, as positive plaques could be easily, rapidly, and preferentially identified with bright-field microscopy according to the images generated by the IncuCyte^®^S3 system. Moreover, the plaques have excellent dispersion in some wells of the six 96-well plates (which is important for the first round of purification) (Figure 3b). This could significantly reduce the rounds of plaque purification, as well as the time per round. The methodology developed by Zong Sheng Guo et al. needs at least ten days to obtain a purified strain of fluorescent rVACV, while ours only needs a maximum of six days. Our method is also an improvement over the EPPIC platform described by Brittany Jasperse et al., as ours does not require any drug-induced selection that may cause unexpected viral genome mutations [16].

Viral plaque counting was based on the chromatic aberration between the healthy cell layers and the lytic lesions, which allowed the identification of plaques by the naked eye. With this in mind, for the non-fluorescent rVACV plaque assay, we constructed two strains of CV-1 stable cell lines expressing either eGFP or TurboFP635. After non-fluorescent rVACV infection, the fluorescence intensity of the infected cells in the formed plaques is much brighter than that of uninfected neighbor cells, ostensibly because of cell–cell fusion triggered by VACV infection; the plaques can be identified via this intensity difference (Figure S5). For the eGFP- and TurboFP635-expressing rVACV, the plaques were detected after being scanned by the IncuCyte^®^S3. This procedure makes the recognizing of plaques easier. In addition, to validate the accuracy of this new approach for VACV titer determination, we also compared the data obtained by crystal violet staining for plaque counting with those of the fluorescence plaque counting by fluorescence-based imaging. Finally, we found that there is no significant difference between these two counting methods (Figure S4). In parallel, we compared the virus plaque numbers of three different strains of TurboFP635-expressing stable monoclonal CV-1 cells with those of the wild-type CV-1 cells. In these experimental settings, the CV-1-Turbo monoclonal cell lines A1 and D2 showed significantly larger plaque numbers compared to the monoclonal cell line F1 and the wild-type CV-1 cells (Figure 9). One possible reason for this phenomenon could be that some VACV replication-related genes were knocked out or down when the lentiviral vector integrated into the host cell genomes to generate the stable cell line. Admittedly, these genes should be further identified and investigated. Of note, we did not find any significant difference between the monoclonal cell line F1 and the wild-type CV-1 cell lines (Figure 9). These results may suggest that our method is suitable for determining the viral titer of non-fluorescent VACV. Furthermore, to facilitate plaque counting, we imported the images of viral plaques into an open-source software, FreeCAD, to count the numbers of plaques, and the images were electronically stored. If the software of the IncuCyte^®^S3 is updated, the analysis with regard to counting the viral plaque number can be undertaken automatically. Recently, Allyson L. Masci and Jorge L. Arias-Arias et al. developed two new and different techniques for the fluorescent plaque assay. The method of the former involved staining cells with fluorescent nucleic acid dyes. The technique of the latter involved staining the cells using fluorescent antibodies. Both utilized an imaging system similar to the IncuCyte^®^S3 [35,36]. However, both methods require cell staining, and the fluorescent nucleic acid dyes as well as the fluorescent antibodies used for cell staining are more expensive than crystal violet. The fluorescence-dependent plaque assay described here not only facilitates VACV titer determination, but also obviates the need to stain with poisonous or expensive chemicals (e.g., crystal violet, DNA dyes, and expensive fluorescent antibodies). Thus, our modified approach is less harmful to researchers’ health, more environmentally friendly, and much more economical.

## 5. Conclusions

Recombinant virus construction and virus titer determination are essential and common experimental techniques in virology and virus research. In this study, we described for the first time a high-throughput approach for fluorescent rVACV generation, together with a rapid viral titer measurement technique, using a multi-well plate imaging system, IncuCyte^®^S3. Construction of rVACV is generally tedious work, and the traditional procedure of positive plaque identification is time-consuming and cumbersome. Compared to the standard viral plaque screening and purification procedure, which uses conventional fluorescence microscopy, our novel experimental strategy allows for the rapid identification of positive plaques and streamlines the generation of recombination vectors. The whole process generally takes six days, with a minimum of just four days, to generate a novel fluorescent rVACV strain, and there is no need for drug-mediated selective pressure or host-range selection (Figure 1). In comparison, the standard process can take up to several weeks [12,37]. The plaque assay is the most widely used method for VACV titer determination. However, the standard procedure of the plaque assay is laborious, time-consuming, and poses environmental health hazards because of the cell layer fixation step and the cell staining chemicals. Here, we outline a fluorescence-dependent plaque assay method for viral titer determination without cell fixation and staining steps by analysis with the IncuCyte^®^S3 system.

In conclusion, our study demonstrates a systematic method of VACV research including both fluorescence recombinant virus construction and titer determination through the use of a multi-well plate imaging system, IncuCyte^®^S3, which makes the research much more effective and easier to undertake. Furthermore, the method described here is optimized for generating rVACV-Lister strains, but it could be helpful for other orthopoxviruses as well. Finally, fluorescence rVACV strains are entering phase III clinical trial and will probably soon be successful [38]. If optimized, our modified virus titer determination methodology can be routinely carried out on tumor samples taken from rVACV-treated cancer patients as well as on liquid biopsies.

## Figures and Tables

**Table 1:** Statistical analysis of eGFP-positive plaques per 96-well plate.

Plate No.	Total Number of Viral Plaques	Number of eGFP-Positive Plaques	Recombination Rate
1	522	3	0.57%
2	550	5	0.91%
3	567	3	0.53%
4	493	4	0.81%
5	487	6	1.23%
6	475	3	0.63%

## Data Availability

The data presented in this study are available on request from the first author.

## Supplementary Materials

The following are available online at https://www.mdpi.com/article/10.3390/biomedicines9081032/s1, Figure S1: Recombinant vaccinia virus L1c-Ig-Turbo, Figure S2: Identification of homologous recombination between the transfected PSG-eGFP plasmid and the vaccinia virus L1c-Ig-Turbo genome after two days of incubation, Figure S3: eGFP-labeled rVACV plaque assay based on wild-type CV-1 cells, Figure S4: Crystal violet-stained manual counting vs. fluorescence-dependent FreeCAD plaque counting, Figure S5: Pixel intensity of Lister 1.1.1-infected fluorescence-labeled stable CV-1 cells. Table S1: Procedure of virus dilution.
