# Supplementary material for: Efficient Selection of Recombinant Fluorescent Vaccinia Virus Strains and Rapid Virus Titer Determination by Using a Multi-Well Plate Imaging System
Source: Biomedicines. 2021 Aug 18;9(8):1032. doi: 10.3390/biomedicines9081032 (PMC8393244; doi:10.3390/biomedicines9081032)
Supplement: Supplementary file 1 [file biomedicines-09-01032-s001.zip › biomedicines-1316992-supplementary.pdf]

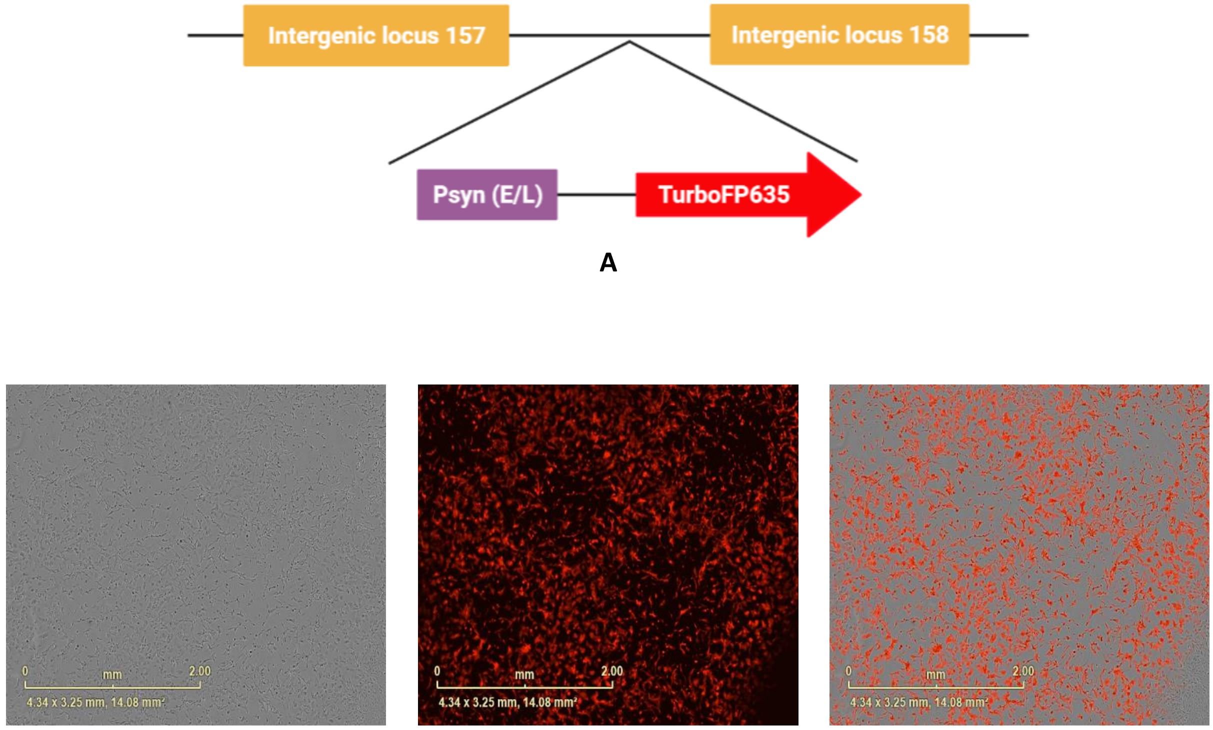

A

B C D

Figure S1. Recombinant vaccinia virus L1c-Ig-Turbo. (**A**) Schematic representation of recombinant vaccinia virus L1c-Ig-Turbo. (**B**) Bright field image of CV-1 cells infected with L1c-Ig-Turbo. (**C**) Fluorescence image of CV-1 cells infected with L1c-Ig-Turbo. (**D**) Overlap of fluorescence image with bright field image. Scale bars represent 2.0 mm.

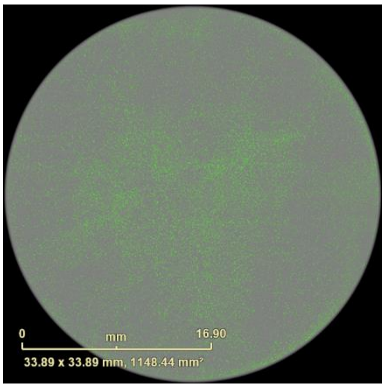

A

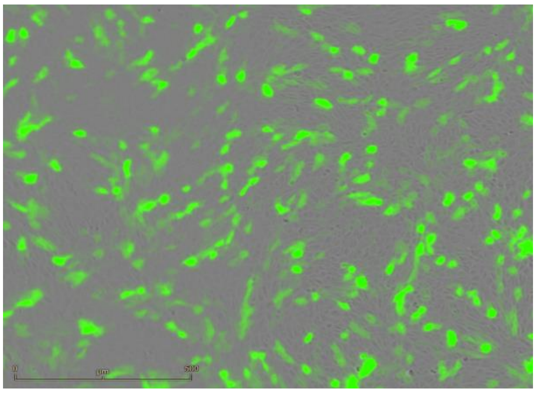

B

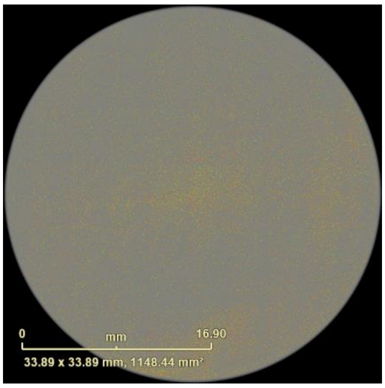

C

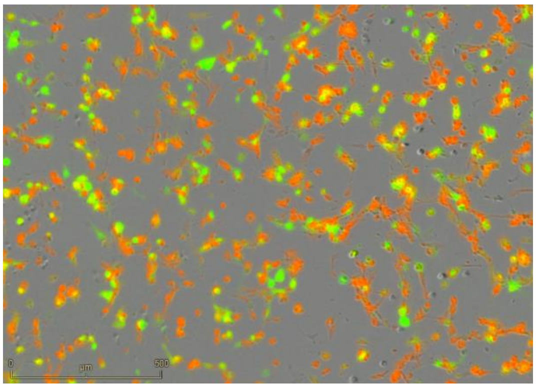

D

Figure S2. Identification of recombinant vaccinia virus L1c-Ig-Turbo-TK-eGFP by fluorescence image (**A-D**). (**A**) Fluorescence image of pEGFP-C2 transfection control well, scale bar=16.9 mm (**B**) Image enlargement (IE) from image (**A**), scale bar=500 µm. (**C**) Homologous recombination between the transfected PSG-eGFP plasmid and the vaccinia virus L1c-Ig-Turbo genome after two days of incubation, overlap of fluorescence image with bright field image, scale bar=16.9 mm (**D**) IE from image (**C**), scale bar represents 500 µm.

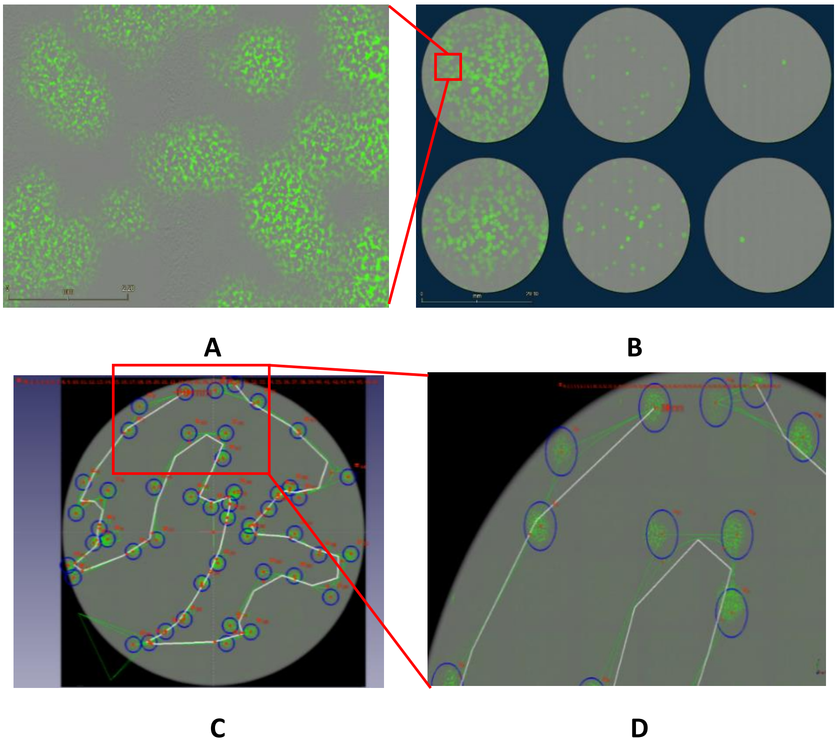

Figure S3. eGFP labelled rVACV plaque assay based on wild type CV-1 cells. Recombinant vaccinia virus GLV-1h109 infected wild type CV-1 cells in 6-well plate (**A-B**). (**A**) IE from (**B**) A1 well, scale bar= 2.2mm. (**B**) GLV-1h109 infected wild type CV-1 cells, scale bar=29.1mm. eGFP labelled viral plaques counted through FreeCAD software (**C-D**). (**C**) Viral plaques counting with GLV-1h109 infected wild type CV-1 cells through FreeCAD software. (**D**) IE from (**C**).

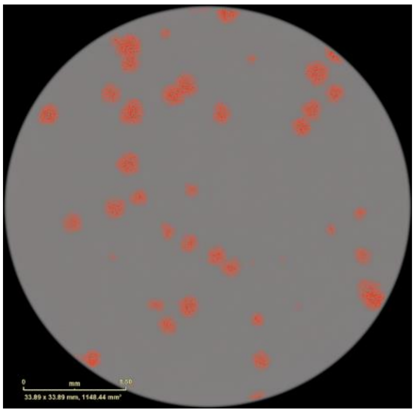

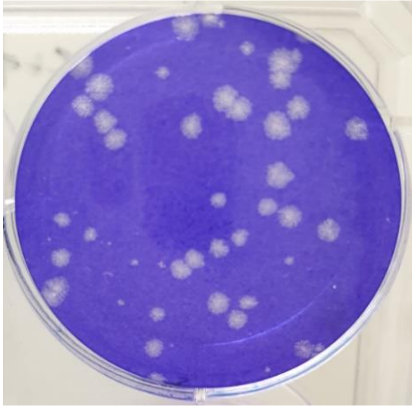

A

B

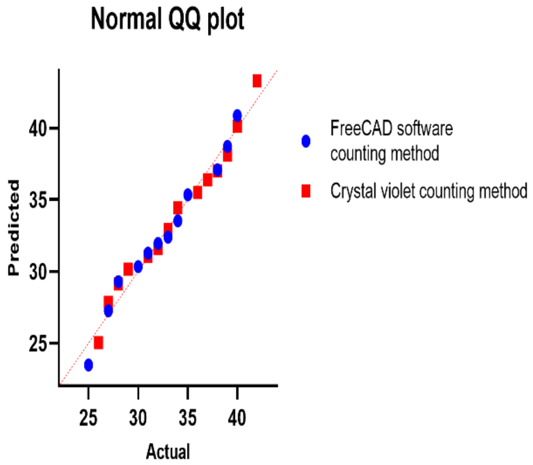

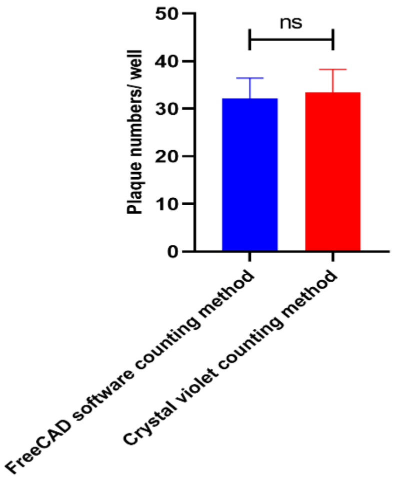

C

D

Figure S4. Crystal violet-stained manual counting vs. fluorescence-dependent FreeCAD plaque counting (**A-D**). (**A**) Viral plaques with L1c-Ig-Turbo-infected CV-1 cell line scanned by Incucyte®S3. (**B**) Crystal violet stain of (**A**). (**C**) Q-Q plot of FreeCAD software and crystal violet counting method. (**D**) Statistical comparison of FreeCAD software and crystal violet counting method (n=28), ns: not significant, P= 0.3479.

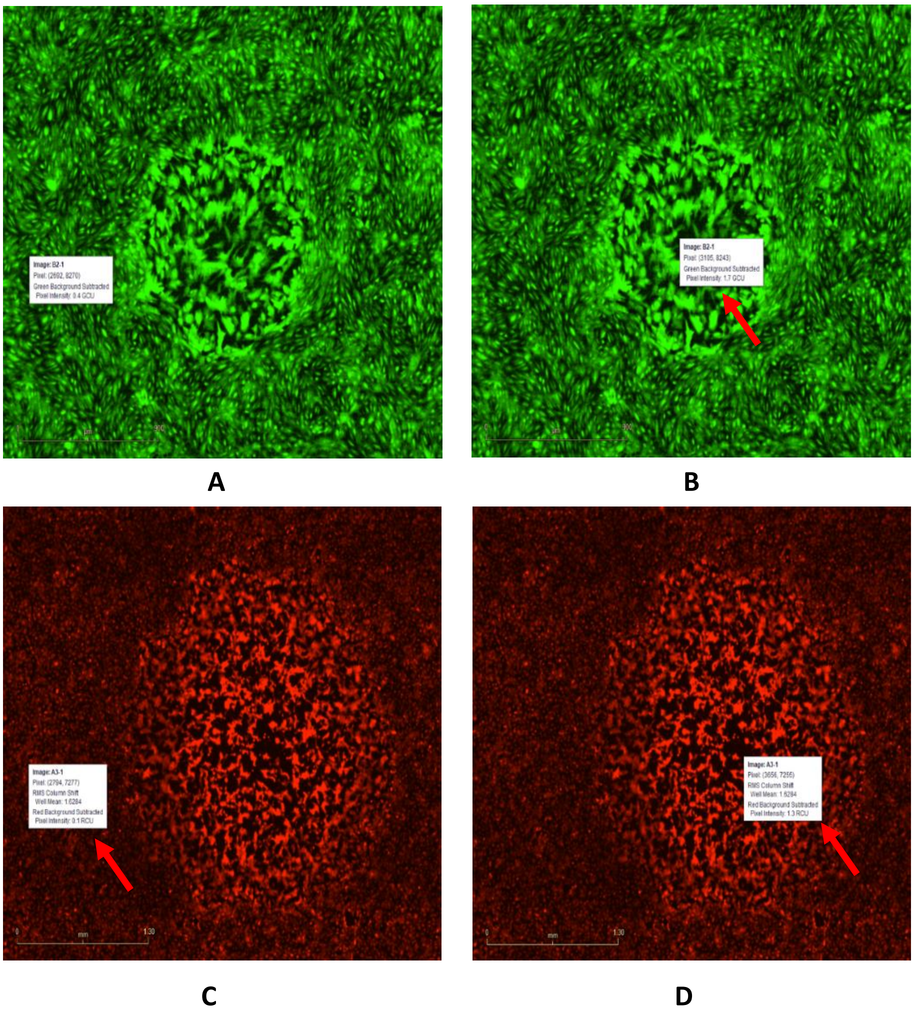

Figure S5. Pixel Intensity of Lister 1.1.1 infected fluorescence labelled stable CV-1 cells. (**A-B**) Lister 1.1.1 infected eGFP labelled stable CV-1 cells. (**C-D**) Lister 1.1.1 infected turboFP635 labelled stable CV-1 cells. (**A**) Green Background Subtracted Pixel Intensity of VACV uninfected cells: 0.4 GCU (**B**) Green Background Subtracted Pixel Intensity of VACV infected cells: 1.7 GCU. (**C**): Red Background Subtracted Pixel Intensity of VACV uninfected cells: 0.1 RCU. (**D**): Red Background Subtracted Pixel Intensity of VACV infected cells: 1.3 RCU.

Table S1. Recipe of virus dilution

Dilutions	Recipe	
10^{-2} dilution	5 µl from virus stock	495 µl DMEM-5% FBS
10^{-3} dilution	100 µl from dilution 10^{-2}	900 µl DMEM-5% FBS
10^{-4} dilution	100 µl from dilution 10^{-3}	900 µl DMEM-5% FBS
10^{-5} dilution	100 µl from dilution 10^{-4}	900 µl DMEM-5% FBS
10^{-6} dilution	100 µl from dilution 10^{-5}	900 µl DMEM-5% FBS
10^{-7} dilution	100 µl from dilution 10^{-6}	900 µl DMEM-5% FBS
10^{-8} dilution	100 µl from dilution 10^{-7}	900 µl DMEM-5% FBS
